# Does Emotional Intelligence of Dental Undergraduates Influence Their Patient Satisfaction?

**DOI:** 10.1155/2021/4573459

**Published:** 2021-09-24

**Authors:** Mandakini Mohan, Kah Heng Lin, Abhishek Parolia, Allan Pau

**Affiliations:** ^1^School of Dentistry, International Medical University, Jalan Jalil Perkasa 19, Bukit Jalil, Kuala Lumpur 57000, Malaysia; ^2^Private Practitioner, Kuala Lumpur, Malaysia

## Abstract

**Objectives:**

The research aimed to investigate if emotional intelligence (EI) scores of dental undergraduates influenced their patients' satisfaction with the treatment received.

**Methods:**

A 33-item EI questionnaire was completed by 46 dental undergraduates in a cross-sectional study. Responses, measured on a five-point Likert scale, were summed to yield EI scores. Patients treated by the same undergraduates were invited to complete a patient satisfaction (PS) questionnaire. EI and PS scores were calculated and compared by undergraduates' gender and the patients' age and education status. The four EI factors (optimism/mood regulation, appraisal of emotions, utilization of emotions, and social skills of students) were correlated with PS using Spearman's correlation test with a significance level set at *p* < 0.05.

**Results:**

EI scores did not differ significantly between male (*N* = 23) and female (*N* = 23) undergraduates (*p*=0.218). PS was not associated with patients' gender, but those educated to the secondary school level were more likely to be satisfied compared to those educated to the college/university level (*p*=0.022). Of the four EI factors, optimism/mood regulation was positively correlated with PS (*p*=0.049).

**Conclusion:**

The results of the study suggest that the EI of the students can influence PS. *Practical Implications*. Interventions to enhance EI can be developed to improve the patient experience.

## 1. Introduction

Quality measurements in healthcare programs are critical as they play a significant role in the outcome, cost of healthcare, and consumers' information and choices. Patient satisfaction is an important and commonly used indicator for measuring the quality of the healthcare provided [1].

Satisfaction is described as an attitudinal response that defines the extent of an individual's experience compared with their expectations [2]. Patient satisfaction is related to the extent to which healthcare needs and expectations of the patient are met [3]. In a review of studies conducted on patient satisfaction, it was proposed that satisfied patients are less likely to change their healthcare provider and more likely to continue using health services and comply with the treatment. Five factors that affect patient's satisfaction with dental care have been identified such as technical competence, interpersonal factors, convenience, costs, and facilities [4]. The dentist–patient relationship is an important provider-controlled factor that determines the overall satisfaction with care received [5–7]. A healthy interpersonal relationship between healthcare professionals and their patients is reported to develop through emotional and social competence [8, 9]. Research suggests that patient satisfaction is positively associated with physicians' ability to respond to the patient's emotions [10, 11], that is, patients are more likely to be satisfied if they had been treated by physicians with higher emotional intelligence (EI) scores.

The concept of EI was introduced as a subset of social intelligence that involves a particular individual monitoring their own and other's emotions, to differentiate among them and use that information to guide their thinking and actions [12]. Although various models of EI such as the trait model, ability model, and the mixed model have since been defined, it is generally agreed that the EI is a multidimensional construct with both cognitive and affective elements [13, 14]. It primarily relates to an individual's personal and social competencies. EI has been reported to positively influence academic performance, work attitudes, work performance, career commitment, and leadership skills [15]. For this reason, it is being considerably used by organizations and academic institutions for recruitment purposes [16]. In the health profession, high EI scores have been linked to improved academic performance [9, 17, 18] and greater stress coping ability [18–20] as well as higher patient–doctor relationship [21] and patient satisfaction [11, 22]. In addition, EI has also been reported to play a role in burnout in health professionals by influencing the personal accomplishment factor [23].

Various measures of EI have been tested and validated by researchers to come to these conclusions [24]. Schutte et al. [14] developed a 33-item scale to assess EI in 1998. Items of this Schutte Self-Report Emotional Intelligence Test (SSEIT) relate to the appraisal and expression of emotion, regulation of emotion, and utilization of emotion. Factor analyses of the 33 items on the scale have resulted in the following four factors: optimism/mood regulation—the ability to maintain a positive emotional outlook or to control emotions when under pressure; utilization of emotions—the ability to use the emotional impact of major events to guide personal development; appraisal of emotions—the ability to recognize; and perceive emotions in self and others and social skills—the ability to empathize and relate with other people [25, 26].

Though EI has been extensively studied, very little information is available on its role in dentistry. Dental undergraduates with higher EI scores have been observed to manage academic and other stress better, leading to improved academic performance [27–29]. There is some indication that EI scores of dental undergraduates influence patient satisfaction [30]. Achieving patient satisfaction becomes even more critical in a dental school setting where the administrators must strike a balance between meeting the needs of the dental undergraduates and patients. Therefore, the present study was aimed to test the hypothesis that the EI of dental undergraduates in a dental school is positively correlated with patient satisfaction. The null hypothesis proposed for the study is that EI of dental undergraduates is not related to their patient satisfaction.

## 2. Materials and Methods

A cross-sectional study was conducted at a private Malaysian university in which the EI of fourth- and fifth-year dental undergraduates of a five-year program was measured and correlated to their patients' self-reported satisfaction with the treatment they had received from the undergraduates.

No sampling method was applied for this study. All the 46 undergraduate students enrolled in years four and five and the patients treated by them during the duration of the study were invited to participate. Following institutional ethics approval (no. BDS I1-11(13)2014), the 46 students in years four and five were briefed verbally at the end of a teaching session. One researcher (KHL) explained the purpose of the research to the potential participants and informed that taking part in the research would require them to complete a 33-item EI questionnaire [14]. All undergraduate participants were also asked to inform the researcher after completing their patients' treatment so that he could approach those patients to collect data on their satisfaction with the treatment received.

The undergraduate students who participated in the research were from the senior cohorts with maximum patient contact. The students were assured of confidentiality, and written consent was taken. Demographic data on cohort, gender, and age were collected from the participating students. Thereafter, the undergraduates completed the Schutte Self-Report Emotional Intelligence Test (SSEIT) on paper by rating the extent to which they agreed or disagreed with each statement. The SSEIT was originally developed in English but has been validated in different populations across various professions [19, 23, 28, 31]. The EI scale comprises 33 items, three of which are reverse-scored, measured on a five-point Likert scale from 1 strongly disagree to 5 strongly agree. This scale has good internal consistency and reliability, with Cronbach's alpha of 0.90, and has fair stability over time, with a 2-week test-retest reliability score of 0.78 [14]. A total EI score was derived by summing up the item responses. The possible scores range from 33, indicating the lowest EI, to 165, indicating the highest EI. Factor analyses of these 33 items performed by previous researchers had identified four factors [25, 26], which were: optimism/mood regulation, utilization of emotions, appraisal of emotions, and social skills. The scores of the items under each of these four factors were summed to obtain the factor scores.

Patients who had received routine dental treatment from the undergraduate participants were approached by a researcher (KHL) for inviting them to participate in this study. Potential patient participants were informed about the purpose of the research and the need to complete a patient satisfaction questionnaire. Participants were assured about the confidentiality of the information, and written consent was obtained from participants who agreed to take part. They were then given the questionnaire for self-completion and advised to place the completed questionnaire in a brown envelope, seal, and return it to the receptionist in the waiting room. The questionnaire, comprising eight questions on a 5-point Likert scale, from 1 strongly disagree to 5 strongly agree, was adapted from a self-administered Dental Satisfaction Questionnaire developed by Davies and Ware [32]. This questionnaire is routinely used in the oral health clinic of the university. Information on the patient's age, gender, ethnicity, and education status were also obtained.

Data were collected from the two self-administered questionnaires and tabulated and analyzed using the IBM SPSS Statistics for Windows, Version 24.0 (IBM Corp., Armonk, NY). Frequency distributions were observed for the outcome and independent variables. EI and PS were tested for association with students and patients' sociodemographic characteristics using the independent sample *t*-test. Students' EI and its four factors, that is, optimism/mood regulation, utilization of emotions, appraisal of emotions, and social skills, were tested for association with PS using the Spearman's correlation coefficient test with significance level set at *p* < 0.05.

## 3. Results

All the 46 undergraduate students enrolled in years 4 and 5 of the dental program participated in the study. Of these 46 participants, 23 (50%) were male, and 23 (50%) were female. Male undergraduates' mean EI score (126.9; 95% CI: 122.2–131.6) was not statistically significantly different from female undergraduates' mean EI score (123.4, 95% CI: 120.0–126.7; *p*=0.218). Mean scores of male undergraduates for each of the four factors were not statistically significantly different to mean scores of female undergraduates. Of the 46 undergraduates, 22 (47.8%) were in year 5 and 24 (52.2%) in year 4. Year 5 undergraduates' mean EI score (124.1, 95% CI: 120.6–127.5) was not statistically significantly different from year 4 undergraduates' mean EI score (126.1, 95% CI: 121.5–130.7; *p*=0.464). Mean scores of year 5 undergraduates for each of the four factors were not statistically significantly different from mean scores of year 4 undergraduates (factor 1, *p*=0.806, factor 2, *p*=0.490, factor 3, *p*=0.174, and factor 4, *p*=0.107; Table 1).

A total of 91 volunteer patients, aged 21 to 75 years, received treatment procedures from the 46 students. There were variable patient responses per student as some students had 1 patient response while others had more than 1 patient who responded. Treatment provided included restorations, extractions, root canal therapy, removable and fixed dental prosthesis, minor oral surgery, bleaching, and root planning over the study duration of 4 months. The respondents included 35 (38.5%) male and 56 (61.5%) female patients. The mean PS score of male patients (36.4, 95% CI: 35.1–37.7) was almost the same as of female patients (36.5, 95% CI: 35.5–37.4) with no significant difference (*p*=0.437). The age of the patients was dichotomized at the median. Fifty-three (58.2%) patients were categorized into those younger than 50 years, while 38 (41.8%) were categorized into those aged 51 years or older. The mean PS score of older patients (37.0, 95% CI: 36.0–38.1) was not statistically significantly different from the mean PS score (36.0, 95% CI: 34.9–37.1; *p*=0.722) of younger patients. Forty-five patients (49.5%) reported their education level as “up to secondary level,” while 46 patients (50.5%) reported their education level as “tertiary.” Patients educated up to the secondary level reported a statistically significantly higher mean PS score (37.3, 95% CI: 36.2–38.4) when compared to those educated up to college or university level (36.0, 95% CI: 34.5–36.6; *p*=0.022; Table 2).

The mean PS score of patients treated by male undergraduates (36.8, 95% CI: 35.8–37.7) was not statistically significantly different from that of patients treated by female undergraduates (36.0, 95% CI: 34.8–37.2; *p*=0.396). The mean PS score of patients treated by year 5 dental undergraduates (37.2, 95% CI: 36.0–38.5) was not statistically significantly different from that of patients treated by year 4 dental undergraduates (36.0, 95% CI: 35.0–37.0; *p*=0.113). PS was statistically significantly correlated to factor 1 (optimism/mood regulation, *p*=0.049, but not to the other three factors (Table 3).

## 4. Discussion

The present study was conducted to evaluate the effect of EI scores of dental undergraduates of years 4 and 5 on the satisfaction scores of treatment received by the patients they treated during the duration of the study. The key finding was that patients treated by dental undergraduates who scored higher on the optimism/mood regulation factor of the Schutte EI scale were more satisfied with the treatment they received compared to those who scored lower.

In the present study, the EI of male students was not significantly different from the EI of female students. Based on the results of previous studies, the gender difference in EI has shown to be inconclusive and variable. Though there is evidence that males display a higher self-perceived EI score than females [30, 33], several other studies have concluded that females report significantly higher EI [27, 29]. Studies have also reported that females are more likely to express positive and negative emotions fluently and frequently, and they are considered to have more interpersonal competencies and therefore more socially skilled and emotional than men [34]. The dental undergraduates of years 4 and 5 in the present study did not have much variation in their age and did not show any significant difference in their EI scores. Similar findings were reported by other researchers who concluded that age is relevant to the evolution of EI [21, 35, 36]. It is an important sociodemographic variable that facilitates the relation between gender and EI in such a way that these differences may diminish or dissipate altogether [34, 36].

Mean scores of patient satisfaction obtained from the 91 patients were almost similar for both male and female patients showing that gender did not influence it significantly. This corresponds to the results of other studies [37, 38]. In the present study, though patients older than 50 years of age did not show significantly higher satisfaction levels than younger patients, but it has been shown in previous studies conducted in hospital setup that people become less critical of healthcare services as they get older [1, 37, 39–41]. This may be attributed to the more realistic expectations of older people towards the healthcare system and professionals based on their experiences. Patients who were educated with a university or college degree had significantly lower satisfaction scores. This could be most likely due to the higher expectations and awareness of the educated person regarding the services provided to them as observed in a previous study [37].

Patient satisfaction is multifactorial and influenced by the physician, patient, and organization [1], and several theories have been proposed to rationalize the effect of patient expectation on satisfaction [3]. However, communication is reported as a crucial factor that may mitigate the effects of a negative experience and enable the development of a positive relationship between the healthcare provider and their patient [42]. Since this competency of communication skills is positively guided by the EI of an individual [43], the present study aimed to evaluate how EI was related to patient satisfaction. A four-factor structure obtained by Petrides and Furnham [33] was utilized in the current study for the EI scale. The relation of the students' EI and patient satisfaction scores revealed that patients treated by dental undergraduates who scored higher on the optimism/mood regulation factor of the EI construct were more likely to be satisfied compared to those treated by dental undergraduates with lower scores. The other three factors analyzed did not show any significant differences in the patient satisfaction scores obtained. Previous studies conducted on physicians have found mood (optimism/happiness) to be the factor that most likely influences patient satisfaction [11]. A statistically significant relationship between the general emotional intelligence score of dental undergraduates and patient satisfaction has been reported by Azimi et al. [30] similar to the findings of the current study conducted on dental undergraduates.

The findings of our study point towards a positive influence of at least one realm of the EI construct on patient satisfaction. These findings, however, may not be definitive due to some inherent limitations in the study. Firstly, the patients included in the study may have felt inclined to give positive comments possibly because they were aware that the competence levels of students may be different from that of dentists. Secondly, the participating students knew about the satisfaction survey and a possible Hawthorne effect may have come into play with the students being consciously pleasant to the patients during the span of the study [21]. In addition, the sociodemographics of the participants of our study are quite similar. This could be a potential cause of bias as well. Finally, the low sample size of students and the variable patient responses per student may also contribute to a limitation of this present study. Nonetheless, further validated research, based on the findings above, can be undertaken to evaluate the relationship of patient satisfaction with EI in a broader setup including other health professions as well. Findings of such research will apprise us of how EI can be realistically used in occupational situations such as for student selection or staff recruitment in healthcare. Furthermore, interventions to enhance EI through programs have been reported [44, 45], but future research could focus on the form of training suitable for a certain population and focusing on specific domains of emotional intelligence. Additionally, factors influencing patient satisfaction other than those related to the physician's personality are of importance too and cannot be disregarded especially as internet-based health services gain acceptance [46] and paves way for expanded patient access to information [47].

## 5. Conclusion

The results of the study suggest that patients treated by undergraduates scoring higher on optimism/mood regulation were more likely to be satisfied than those treated by undergraduates scoring lower, thereby implying that this dimension of EI is associated with PS.

The current study was conducted on a limited group of dental undergraduates displaying very similar social demographics and possibly social experiences. This would be the most likely limitation of the study along with the small sample size of the patients. This study however offers an interesting insight into the positive significant correlation between one factor of EI and the satisfaction levels of patients. It would be useful to see how interventions may enhance the EI scores of individuals to help them perform better in their professional and personal skills.

## Figures and Tables

**Table 1:** Comparison of mean EI and factor scores of dental undergraduate's gender and year of the study.

	*N* (%)	EI (mean, 95% CI)	*p* value	Factor 1^*∗*^ (mean, 95% CI)	*p* value	Factor 2^*∗*^ (mean, 95% CI)	*p* value	Factor 3^*∗*^ (mean, 95% CI)	*p* value	Factor 4^*∗*^ (mean, 95% CI)	*p* value
Gender											
Male	23 (50.0)	126.9 (122.2–131.6)	0.218	47.6 (45.4–49.7)	0.117	22.4 (21.1–23.6)	0.495	23.0 (21.8–24.3)	0.903	33.9 (32.9–35.0)	0.422
Female	23 (50.0)	123.4 (120.0–126.7)		45.3 (43.4–47.3)		21.8 (20.7–22.9)		22.9 (22.2–23.7)		33.3 (32.0–34.6)	

Cohort											
Year 5	22 (47.8)	124.1 (120.6–127.5)	0.464	46.3 (44.6–47.9)	0.806	22.4 (21.3–23.4)	0.490	22.5 (21.4–23.6)	0.174	32.9 (31.8–34.1)	0.107
Year 4	24 (52.2)	126.1 (121.5–130.7)		46.6 (44.2–49.0)		21.8 (20.5–23.1)		23.5 (22.5–24.4)		34.3 (33.1–35.4)	

^*∗*^Factor 1: optimism/mood regulation, factor 2: utilization of emotions, factor 3: appraisal of emotions, and factor 4: social skills.

**Table 2:** Association between patient's gender, age, and education level with their satisfaction scores.

		*N* (%)	PS score mean (95% CI)	*p* value
Gender	Male	35 (38.5)	36.4 (35.1–37.7)	0.437
	Female	56 (61.5)	36.5 (35.5–37.4)	

Age	50 years and younger	53 (58.2)	36 (34.9–37.1)	0.722
	51 years and older	38 (41.8)	37 (36–38.1)	

Education	Up to secondary	45 (49.5)	37.3 (36.2–38.4)	0.022
	Tertiary	46 (50.5)	36.0 (34.5–36.6)	

**Table 3:** Association between dental undergraduate's gender, year of the study, EI scores, and PS.

		Mean PS (95% CI)	*p* value

Student's gender	Male	36.8 (35.8–37.7)	0.396
	Female	36.0 (34.8–37.2)	

Year of study	Year 4	36.0 (35–37)	0.113
	Year 5	37.2 (36–38.5)	

		Spearman coefficient	*p* value
EI factors	Factor 1	0.175	0.049
	Factor 2	0.013	0.450
	Factor 3	0.162	0.062
	Factor 4	0.010	0.462

## Data Availability

All the relevant data are included within the paper. Additional data are available upon request to the corresponding author.
